# External Load, More than Surface Instability, Drives Post-Activation Performance Enhancement in Split Squat Conditioning Activity: Phase-Specific EMG Responses

**DOI:** 10.3390/jcm15082927

**Published:** 2026-04-12

**Authors:** Jinyong Sim, Hanbee Jang, Yujin Jeong, Sanghee Park

**Affiliations:** 1 The Integrative Movement Science Laboratory, Gachon University, Incheon 21936, Republic of Korea; sjy5280@gachon.ac.kr (J.S.); jhb05@gachon.ac.kr (H.J.); 17rachel@gachon.ac.kr (Y.J.); 2 Department of Exercise Rehabilitation, Gachon University, Incheon 21936, Republic of Korea

**Keywords:** PAPE, split squat, BOSU, conditioning activity, electromyography, functional movement, vertical jump

## Abstract

**Background:** Conditioning activity (CA) is used to elicit post-activation performance enhancement (PAPE), but it is unclear whether load response principles from back squat models generalize to unilateral split squat conditioning when external load and surface instability are manipulated together. Thus, the current study examined acute effects of stable vs. unstable split squat CA with or without external load on jump performance and phase-specific electromyography (EMG). **Methods**: Twenty men completed a randomized crossover of three CAs (2 × 3 reps): unloaded stable split squat (SS), unloaded BOSU SS, and BOSU loaded at 50% split squat one-repetition maximum. Single leg jump (SLJ) and countermovement jump (CMJ) were assessed pre-CA and at 3 min (SLJ) and 4 min (CMJ) post-CA. EMG was recorded from the biceps femoris (BF), semitendinosus (ST), vastus lateralis (VL), vastus medialis (VM) gluteus medius (Gmed), peroneus longus (PL), gastrocnemius lateralis (GL) and gastrocnemius medialis (GM). Signals were time-normalized across the split squat cycle and quantified using phase-specific area under the curve (AUC) (descending/ascending). **Results:** SLJ and CMJ increased after all conditions compared with the pre-test (*p* < 0.05). SS and unloaded BOSU SS produced comparable jump outcomes, whereas BOSU loaded yielded the greatest CMJ increase (*p* < 0.04). Unloaded BOSU SS selectively increased hamstring activation (BF, ST) without changes in Gmed or PL. BOSU loaded increased EMG amplitude across all measured muscles. **Conclusions:** External load primarily drives acute CMJ potentiation, whereas instability mainly redistributes recruitment toward the hamstrings without improving jump performance beyond the stable condition. These findings indicated that when the goal is acute jump enhancement, external load should be prioritized, whereas unstable surfaces may be used to selectively target posterior chain activation.

## 1. Introduction

Explosive power, defined as the ability to rapidly generate force, is a key determinant of competitive performance across many sports. Accordingly, coaches and researchers continuously seek both chronic training strategies and acute warm-up methods that can meaningfully enhance subsequent power-related tasks (e.g., jumping, sprinting, and rapid directional changes) [1,2]. A widely used approach is the inclusion of conditioning activities during warm-up, where post-activation performance enhancement refers to an acute increase in neuromuscular capacity following a brief bout of isotonic or isometric exercise performed before an explosive task [3,4,5]. Proposed contributors include enhanced muscle activation and increased muscle-tendon stiffness, which may transiently improve force production and rate of force development [3]. In parallel, classic potentiation-related mechanisms include increased phosphorylation of myosin regulatory light chains and increased motor unit recruitment, which can be indirectly reflected in electromyographic measures, ultimately elevating muscle tension and strength/power expression [1].

Importantly, vertical jump performance, particularly CMJ and SLJ, has been widely adopted as a sensitive and reliable indicator of PAPE responses due to its strong association with lower-limb explosive power and rate of force development [6,7,8]. CMJ reflects coordinated force production across the entire lower-limb kinetic chain under bilateral conditions, whereas SLJ provides additional insight into unilateral force expression and inter-limb neuromuscular control [3,9]. Therefore, the combined use of CMJ and SLJ allows a more comprehensive evaluation of whether conditioning activities translate into functionally meaningful performance enhancement. In parallel, EMG analysis offers mechanistic insight into the neuromuscular strategies underlying performance changes following conditioning activities. Phase-specific EMG quantification, particularly using time-normalized signals and AUC, enables the identification of how muscle activation is redistributed across movement phases (e.g., eccentric and concentric phases) rather than relying solely on peak amplitude measures [10]. This approach is particularly relevant in complex, multi-joint tasks such as the split squat where coordination and timing of muscle activation may be as important as overall activation magnitude. Consequently, combining jump performance outcomes with phase-specific EMG analysis provides a more comprehensive framework for interpreting both the effectiveness and the underlying mechanisms of PAPE interventions.

Traditionally, most PAPE protocols have emphasized bilateral squat-based CAs. However, many sport actions are executed under unilateral loading patterns and single-limb force production, where postural control and coordination become critical performance factors. Consequently, unilateral resistance exercises such as the split squat, Bulgarian split squat (BSS) and single-leg squat are frequently utilized to develop unilateral strength and dynamic stability in a manner that better reflects sport-specific demands [11]. Nevertheless, PAPE outcomes are not consistently observed even when CAs appear biomechanically similar to the target task. For instance, Papla et al. reported that both bilateral and unilateral complex CAs (back squat + drop jumps vs. split squat + depth jumps to lateral hop) failed to produce significant improvements in countermovement jump or change-of-direction performance [12]. These findings underscore that movement pattern similarity alone does not guarantee measurable performance enhancement. At the same time, other studies [5,13] have reported improved jump performance following seemingly comparable squat-based conditioning, suggesting that the PAPE response may depend on the interaction between external load application and how the movement is manipulated (e.g., unilateral vs. bilateral execution or stability demands), rather than on exercise selection alone.

With respect to external load, substantial effort has focused on optimizing CA “dose,” particularly intensity (% one repetition maximum (%1RM)) and the interval between CA and performance testing. In back squat-based protocols, a reasonable consensus indicates that moderate to high intensities combined with rest intervals of approximately 2–12 min can facilitate detectable PAPE in jump-based outcomes [14,15,16]. However, compared with the extensive back squat literature, evidence examining external load application for unilateral conditioning including split squat-based protocols remains limited, despite their superior feasibility in field environments (e.g., kettlebell loading and minimal equipment requirements). As a result, it remains unclear whether dose response principles derived from back squat models generalize to split squat conditioning, particularly when external load is applied.

In addition to load prescription, movement manipulation strategies aimed at augmenting neuromuscular activation have increasingly incorporated instability-based tools (e.g., BOSU and suspension systems) into unilateral training [2,11]. However, neuromuscular responses to instability are not uniform; they appear highly dependent on how instability is applied (e.g., rear-foot suspension [17], surface compliance [18]), where it is applied (e.g., rear vs. front support [6,18] or BOSU orientation [19]) and whether external load is concurrently imposed [15,16]. Recent work on BSS tasks indicates that instability configuration can alter lower-limb muscle activation patterns even without external loading, highlighting surface selection as a meaningful programming variable [18]. At the distal level, BOSU-based single leg tasks have also been associated with increased stabilizer demands (e.g., gluteus medius and/or peroneus longus activity) relative to stable ground, supporting a plausible pathway through which instability could influence neuromuscular stimulation and take-off control [18,20]. From a performance perspective, however, chronic comparisons of stable vs. unstable plyometric training often show similar jump improvements across conditions, sustaining debate as to whether instability meaningfully enhances power output or primarily redistributes control demands toward stabilization [2]. Therefore, direct evidence remains limited regarding whether a loaded split squat CA performed on an unstable surface can acutely potentiate jump performance, and whether any potentiation is accompanied by distinct, task-relevant muscle activation patterns.

Accordingly, the current study examined the acute effects of a loaded split squat CA performed on an unstable surface on single leg and countermovement jump performance and assessed associated phase-specific changes in muscle activation. We hypothesized that external load would be the primary driver of PAPE, resulting in measurable improvements in jump performance. In contrast, surface instability was expected to primarily influence neuromuscular activation patterns, leading to selective increases in stabilizer and posterior chain muscle activity without significant additional improvements in jump performance.

## 2. Materials and Methods

### 2.1. Subject Information

Twenty-five recreationally active men were recruited between September 2025 and February 2026. Three participants were excluded according to predefined criteria (i.e., meeting family history relevant to cardiovascular issues or injury-related criteria) and two withdrew before completing the experimental visit due to schedule conflicts or minor illness. The final sample comprised twenty males (age: 24.2 ± 5.9 years; height: 173.9 ± 0.9 cm; body mass: 76.6 ± 8.2 kg). All participants received written and verbal explanations of study procedures and provided informed consent prior to participation. Inclusion criteria were as follows: (1) recreationally active status (≥2–3 sessions/week of structured physical activity for ≥6 months), and (2) ability to perform split squat with standardized technique. Exclusion criteria were as follows: (1) any diagnosed neurological disorders, (2) current orthopedic pathology including persistent lower back pain or lower-limb musculoskeletal injuries involving the hip, knee, or ankle within the last 6 months which required medical care, and (3) uncontrolled hypertension or other cardiovascular responses to high intensity exercise. This study was approved by the Institutional Review Board of Gachon University (IRB No. 1044396-202502-HR-039-01, 15 April 2025), registered with Clinical Research Information Service (Trial registration No. PRE20250604-008, 4 June 2025) and was conducted in accordance with the Declaration of Helsinki.

### 2.2. Study Design

A randomized crossover design using Latin square/counterbalanced order was implemented to examine PAPE with participants completing SLJ and CMJ tests under three split squat CA conditions: (1) stable surface split squat, unloaded (SS); (2) unstable surface split squat on a BOSU, unloaded (BOSU); and (3) BOSU split squat loaded to 50% of split squat 1RM (BOSU loaded). All experimental testing was completed within a single laboratory visit following four familiarization visits. Each CA consisted of two sets of three repetitions with 1 min rest between sets.

After completing familiarization and the split squat 1RM assessment, participants attended an experimental session. A standardized general warm-up consisting of five dynamic stretches, followed by three maximal SLJ and CMJ attempts with a 15 s inter-trial rest interval. After a 4 min rest period, participants completed pre-test SLJ and CMJ in a consistent manner (three maximal trials each; 15 s rest between trials; fixed order: SLJ followed by CMJ). After a 4 min rest following the pre-test, participants performed two specific warm-up trials and three load-specific trials at low-to-moderate intensity in rehearsal sets designed to standardize movement exposure across conditions and avoid unintended movement pattern from high-intensity efforts.

**Figure 1:**
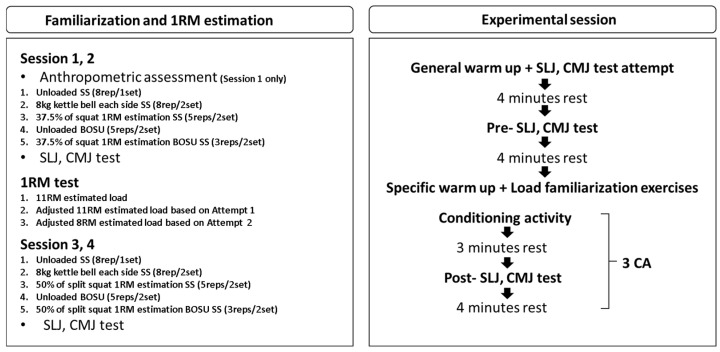
Schematic presentation of the study design.

Participants then completed the randomly assigned CAs: SS (2 × 3 unloaded), BOSU (2 × 3 unloaded), or BOSU loaded (2 × 3 at 50% 1RM). For both BOSU conditions, the BOSU was positioned dome side down (platform side up) to standardize a tolerable instability level; our pilot testing indicated that when the BOSU was positioned dome side up, most participants could not reliably complete three repetitions in BOSU loaded due to balance-related technical failure. Accordingly, BOSU conditions were performed only with the dome-side-down configuration. The 50% 1RM load was selected based on pilot testing as the high tolerable intensity that could be completed on BOSU with acceptable technique and without frequent failure [12]. Post-test SLJ and CMJ were initiated in 3 min and 4 min, respectively, after completion of the CA, consistent with commonly used post-CA time points for capturing PAPE responses [1,14,16,21]. This recovery interval was selected based on previous evidence indicating that PAPE effects can occur within 2–12 min following moderate-to-high intensity conditioning activities, with the most pronounced responses typically observed within the 3–5 min window [14,16]. This procedure was repeated for all three conditions (Figure 1).

### 2.3. Warm-Up and Familiarization

Across all familiarization and experimental visits, a standardized general warm-up was performed: 10 leg swings, 5 repetitions per side of the world’s greatest stretch, 10 forward lunges, 10 jumping jacks, and 10 unloaded squats. Split squat cadence during practice and CAs was standardized using a metronome at 40 bpm (1.5 s eccentric/1.5 s concentric). The specific warm-up consisted of 8 unloaded split squats followed by 2 sets of 8 split squats holding two 8 kg kettlebells with 1 min rest between sets. During familiarization visits 1–2, participants practiced the three CA conditions using loads derived from self-reported back squat 1RM. Based on coaching conventions [22], split squat 1RM was provisionally estimated as approximately 75% of back squat 1RM; therefore, kettlebell loads equivalent to around 37.5% of self-reported back squat 1RM were used for load familiarization in stable and BOSU conditions. During familiarization visits 3–4 after the split squat 1RM assessment, loading was modified so that BOSU-loaded practice used 50% of the estimated split squat 1RM, with sets and repetition held constant. Familiarization with the SLJ and CMJ assessments were conducted after all practice trials.

### 2.4. Determination of Split Squat 1RM

Following the second familiarization session and before the third session, split squat 1RM was assessed using a progressive loading protocol. Because instability-based loading can elevate technical failure risk, an estimated 1RM approach was adopted using multi-repetition maximum attempts within the 8–11RM range on a stable ground, with load adjusted across up to three attempts. In detail, the first and second attempts were performed at an estimated 11RM load, with the second adjusted based on the first 11RM loads. The third attempt was adjusted according to the second and set at an estimated 8RM load based on the preceding 1RM to confirm if the 11RM loads represent true 1RM load with higher loaded condition such as 8RM. Trials were invalidated if cadence deviated substantially from 40 bpm or if balance was lost. Verbal encouragement was provided during each attempt to promote maximal effort.

### 2.5. Anthropometrics and Task Standardization

On the first visit, height, body mass, resting blood pressure, leg length and dominant leg were recorded. Dominant leg was determined based on three functional tasks: (1) preferred kicking leg, (2) the first stepping leg when descending from a box, and (3) the leading leg during stair ascent. The leg identified in at least two of the three tasks was designated as the dominant limb. Leg length was defined as the distance from the anterior superior iliac spine (ASIS) to the medial malleolus. Split squat step length was standardized to 100% (±5%) of leg length and step width was standardized to 75% (±5%) of hip width (the distance between right and left ASIS) [23]. Parameters were refined via pilot tests to ensure suitability for the study population. During all trials, the medial arch of the dominant foot was positioned at the center of the force plate to ensure consistent foot placement and accurate detection of take-off and landing events for jump height analysis. Rear foot placement was standardized according to the predetermined leg length and width with the hallux aligned to a pre-marked reference point to ensure consistent positioning across sessions. The floor was marked to enable consistent rear foot placement at each visit.

### 2.6. Exercise Technique Criteria and Behavioral Controls

All repetitions were performed at a standardized cadence through a consistent range of motion without loss of balance. Movement tempo was controlled using a metronome set at 40 bpm. Each conditioning activity consisted of two sets of three repetitions with a fixed 1 min rest interval between sets. Participants descended until the dominant thigh was approximately parallel to the floor and ascended to full knee extension. A fixation cross was positioned at eye level on the wall in front of the participants to standardize gaze direction. For BOSU conditions, the device was consistently positioned dome side down (platform side up) to ensure a uniform level of instability, as established during pilot testing. To ensure safe and consistent foot placement on the BOSU, the instructor stabilized the BOSU during initial foot contact and withdrew support once balance was achieved. Participants were instructed to maintain their habitual diet and physical activity and to refrain from strenuous exercise, caffeine, and alcohol for at least 48 h before the experimental session.

### 2.7. Outcome Measurement

#### 2.7.1. Measurement of Jumping Performance

Jump height was selected as the primary outcome because it represents a practical and integrative measure of explosive performance. In contrast, force-, power- and rate of force development-related variables were considered secondary mechanistic indicators and were therefore not included in the primary analysis, in line with the applied objective of the current study. All SLJ and CMJ trials were performed on an AMTI force platform (BMS600600-2000, Watertown, MA, USA) interfaced with NetForce software (version 3.5.3; Advanced Mechanical Technology Inc., Watertown, MA, USA), with vertical ground reaction force (vGRF) data sampled at 1000 Hz [6]. The vGRF signal was processed using the manufacturer’s default filtering settings to reduce high-frequency noise. Jump height was calculated using the flight time (FT), defined as the period between take-off and landing, using the following equation, where *g* represents the acceleration of gravity (9.81 m/s^2^) [7]. Take-off was defined as the instant when vGRF fell below 20 N, and landing was identified when vGRF exceeded 20 N [8]. The threshold-based approach has been shown to provide reliable and consistent estimation of jump performance [7,8].

$$Jump\;height=\frac{\left(g\times FT^{2}\right)}{8}$$

For SLJ, participants were instructed to stand upright with their hands placed on the iliac crests to eliminate arm swing contribution. The non-dominant leg was held with the knee slightly flexed and with the foot adjacent to the ankle of the dominant leg; swinging or additional movement of the non-dominant leg was not permitted [9]. Upon a verbal “ready” signal from the investigator, participants stepped on a force platform, and on the signal “go”, participants performed a maximal vertical jump and were instructed to land at approximately the same location on the force platform. For CMJ, participants performed a bilateral vertical jump under an identical procedure except that both limbs were used for take-off and landing; countermovement depth was self-selected [24]. Three maximal trials were collected per test with 15 s inter-trial rest. To ensure consistency and reduce inter-limb variability, SLJ was performed using the dominant leg only while CMJ was assessed bilaterally as a global measure of lower-limb explosive performance. This approach was adopted because the primary aim of this study was to evaluate overall performance enhancement following conditioning activity rather than to investigate inter-limb asymmetry.

#### 2.7.2. Surface EMG

Surface EMG was selected due to its suitability for non-invasive, multi-muscle assessment during dynamic functional tasks involving coordinated lower-limb activity. Muscle activation was recorded using surface electromyography (TELEmyo DTS; Noraxon USA Inc., Scottsdale, AZ, USA) at a sampling frequency of 2000 Hz. EMG signals were band-pass filtered (20–500 Hz; fourth-order Butterworth), full-wave rectified, and processed as root mean square (RMS) using a 100 ms moving window in Noraxon Myo-Research XP Master Edition software (version 4.2.22; Noraxon USA Inc., Scottsdale, AZ, USA). RMS amplitudes were then normalized to each participant’s maximal voluntary isometric contraction (MVIC) and expressed as a percentage of MVIC (%MVIC). Disposable electrodes (Bio-Protech Inc., Tustin, CA, USA) (diameter: 10 mm, bipolar configuration, and an interelectrode distance > 20 mm) were used [20]. On the dominant leg, the surface electrodes were placed on the gluteus medius (Gmed), biceps femoris (BF), semitendinosus (ST), vastus medialis (VM), vastus lateralis (VL), peroneus longus (PL), gastrocnemius medialis (GM), and gastrocnemius lateralis (GL). Prior to the electrode placement, general warm-up was completed and participant’s skin was shaved and cleansed with alcohol to reduce impedance. Electrode sites were located according to SENIAM guidelines, marked for consistency, and leads were secured using elastic adhesive tape to minimize movement artifacts. Light isometric contractions were performed to confirm targeted muscle-specific activation and visually inspect signal quality and crosstalk.

#### 2.7.3. MVIC Procedure

For each muscle, participants performed three 5 s MVIC trials with 30–60 s rest between trials. MVICs were conducted in standardized manual muscle testing positions appropriate for each muscle group (e.g., hip abduction for Gmed, knee flexion for BF and ST, knee extension for VM and VL, ankle plantarflexion for GM and GL, and ankle eversion for PL) following established protocols [25,26]. EMG processing parameters matched those used for CA trials. Verbal encouragement was provided to ensure maximal effort.

### 2.8. Statistical Analyses

Sample size was estimated using G*Power (v3.1; Universität Kiel, Kiel, Germany) for a repeated measure, assuming a two-tailed effect size of 0.5, an alpha level of 0.05, and a statistical power of 0.80, resulting in a required sample size of 20. This estimation was consistent with previous performance studies of similar design [21,27,28]. The selected medium effect size (Cohen’s d = 0.5) was based on prior PAPE and EMG studies reporting moderate changes in jump performance and neuromuscular activation following similar conditioning activities, thereby providing a conservative and methodologically appropriate estimate for the present design [1,26]. This approach ensured adequate statistical power to detect meaningful within-subject changes across conditions. For statistical comparison, one-way repeated measures ANOVA followed by Holm–Sidak multiple comparisons test was applied to evaluate differences in SLJ and CMJ, EMG outcomes across CA conditions. Effect sizes were calculated and reported as partial eta squared (η_p_^2^) for ANOVA and Cohen’s d for pairwise comparisons to quantify the magnitude of observed effects. Data were presented as the mean ± standard error of the mean (SEM), and statistical significance was set at *p* < 0.05. Outliers were handled using a predefined criterion (e.g., >2 standard deviation from the participant’s condition mean). All analyses were conducted using GraphPad Prism (version 11.0.0; GraphPad Software, San Diego, CA, USA).

### 2.9. Use of Generative AI

Generative AI (ChatGPT 5.2) was used solely to improve English language clarity and readability. No AI tools were used to generate, analyze, or modify data, results, or figures.

## 3. Results

Outcomes were analyzed in two domains: jump performance (SLJ and CMJ) following each CA and muscle activation patterns recorded during the CA. Jump performance was expressed as a percentage of the pre-test value (pre-test = 100%). EMG was time-normalized across the split squat cycle and quantified using phase-specific AUC for the descending and ascending phases.

### 3.1. Jump Performance

**Figure 2:**
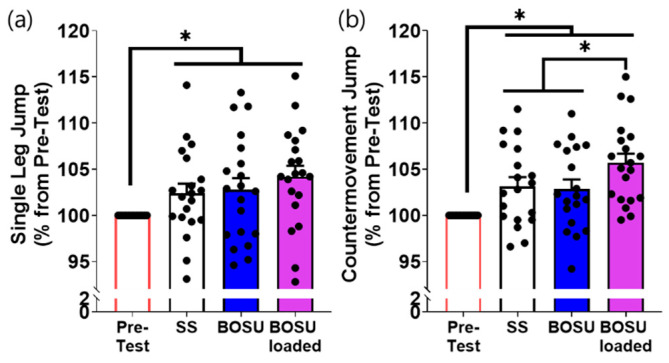
Powerful performance analysis in the comparison from the conditioning activities. (**a**) SLJ, (**b**) CMJ. * indicates statistical significance between the indicated comparison. SS: split squat.

Both SLJ and CMJ increased after each CA relative to the pre-test (*p* < 0.05; Figure 2a,b). When CA was performed without external load, changing the surface (SS vs. BOSU) resulted in comparable outcomes for both SLJ and CMJ. In contrast, adding external load on BOSU (BOSU loaded) produced the largest increase in CMJ and was significantly greater than both SS and BOSU unloaded (*p* < 0.05; η_p_^2^ = 0.32; Figure 2b). For SLJ, BOSU loaded exhibited the highest mean value; however, the differences versus SS and BOSU unloaded were not statistically significant (*p* = 0.20, d = 0.35; *p* = 0.75, d = 0.08, respectively, Figure 2a).

### 3.2. Muscle Activation

**Figure 3:**
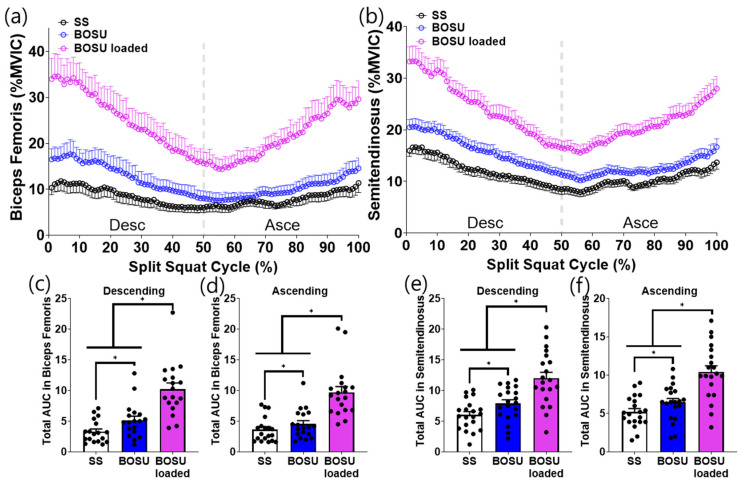
Hamstring muscle activation during the conditioning activities. (**a**,**b**) Averaged linear envelope of biceps femoris and semitendinosus EMG (% mean EMG activities among the conditioning activities) in the split squat cycle. (**c**–**f**) AUC of biceps femoris and semitendinosus activation for descending and ascending cycle, respectively. * indicates statistical significance between the indicated comparison. SS: split squat, AUC: area under the curve.

**Figure 4:**
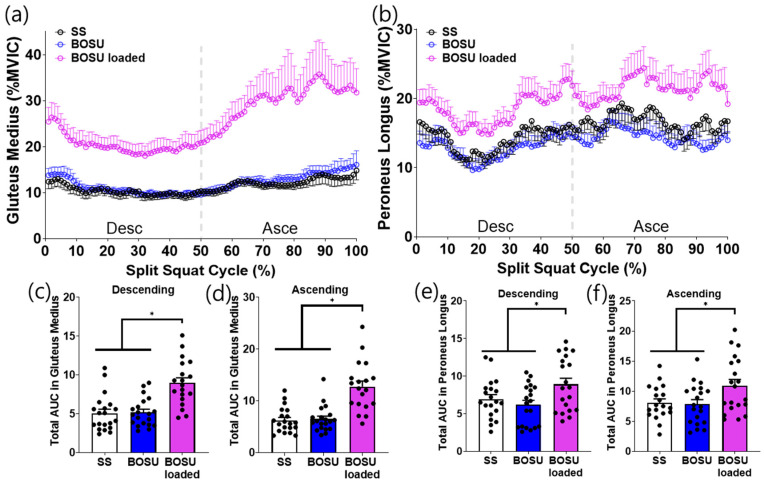
Leg stabilizer muscle activation during the conditioning activities. (**a**,**b**) Averaged linear envelope of gluteus medius and peroneus longus EMG (% mean EMG activities among the conditioning activities) in the split squat cycle. (**c**–**f**) AUC for gluteus medius and peroneus longus activation during descending and ascending cycle, respectively. * indicates statistical significance between the indicated comparison. SS: split squat, AUC: area under the curve.

**Figure 5:**
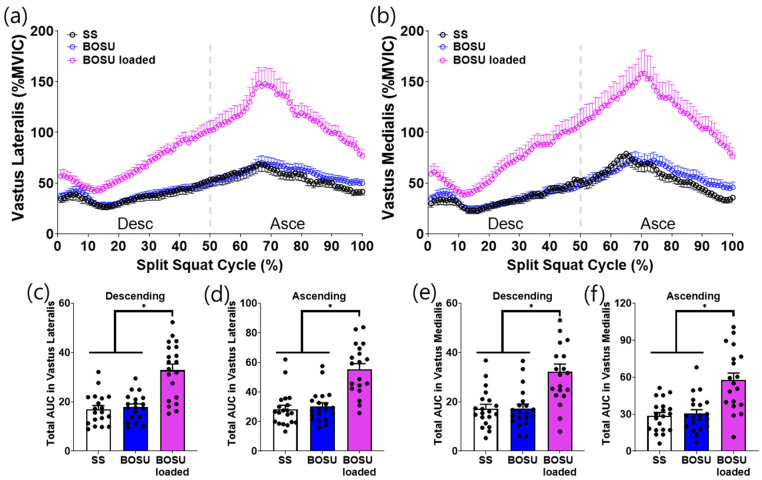
Quadriceps muscle activation during the conditioning activities. (**a**,**b**) Averaged linear envelope of vastus lateralis and vastus medialis EMG (% mean EMG activities among the conditioning activities) in the split squat cycle. (**c***–***f**) AUC of vastus lateralis and vastus medialis activation for descending and ascending cycle, respectively. * indicates statistical significance between the indicated comparison. SS: split squat, AUC: area under the curve.

**Figure 6:**
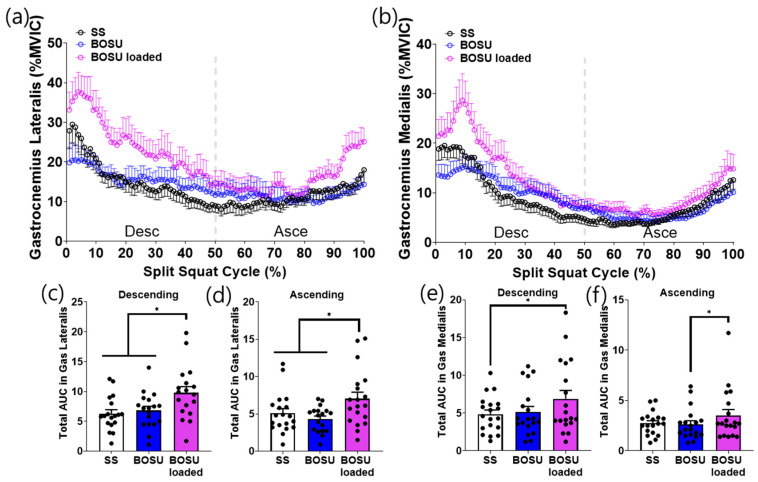
Gastrocnemius muscle activation during the conditioning activities. (**a**,**b**) Averaged linear envelope of gastrocnemius lateralis and gastrocnemius medialis EMG (% mean EMG activities among the conditioning activities) in the split squat cycle. (**c**–**f**) AUC for gastrocnemius lateralis and gastrocnemius medialis activation during descending and ascending cycle, respectively. * indicates statistical significance between the indicated comparison. SS: split squat, AUC: area under the curve.

EMG responses demonstrated a distinct dissociation between surface effects (SS vs. BOSU, both unloaded) and load effects (BOSU loaded vs. unloaded conditions) across muscle groups (Figure 3, Figure 4, Figure 5 and Figure 6).

Surface effect (SS vs. BOSU, both unloaded): A surface change alone selectively increased hamstring activation. Specifically, unloaded BOSU elicited higher biceps femoris and semitendinosus activity than SS across the split squat cycle with corresponding increases in phase-specific AUC during both the descending and ascending phases (*p* < 0.05; Figure 3c–f). In contrast, stabilization-related muscles (gluteus medius and peroneus longus) did not differ between SS and unloaded BOSU in either phase (Figure 4c–f). Quadriceps (vastus lateralis, vastus medialis) and gastrocnemius (lateralis and medialis) also showed no statistically significant differences between SS and unloaded BOSU in phase-specific AUC comparisons (Figure 5c–f and Figure 6c–f).

Load effect (BOSU loaded vs. unloaded conditions): Adding external load on BOSU increased EMG amplitude across all measured lower-limb muscles. BOSU loaded elicited greater activation than both SS and unloaded BOSU for the hamstrings (Figure 3), stabilization-related muscles (Figure 4), quadriceps (Figure 5), and gastrocnemius (Figure 6) with consistent increases in phase-specific AUC across the descending and/or ascending phases (Figure 3, Figure 4, Figure 5 and Figure 6).

## 4. Discussion

The primary finding of the current study is that external loading, rather than foot instability per se, was the dominant determinant of jump potentiation following a split squat conditioning activity while all CAs increased jump performance. Although all CA conditions increased jump performance relative to baseline, the clearest additional improvement was observed in CMJ after the BOSU condition performed at 50% split squat 1RM, whereas changing the surface without load (SS vs. BOSU) provided no meaningful advantage in either CMJ or SLJ. The EMG findings further clarified this dissociation: unloaded BOSU selectively increased hamstring activation, whereas loaded BOSU increased EMG amplitude across all measured lower-limb muscles. Together, these results indicate that a split squat CA can elicit PAPE in a field-feasible setting, and that load prescription primarily amplifies performance gains while instability mainly reshapes the neuromuscular recruitment pattern.

### 4.1. PAPE Effect

This load-dominant performance response is consistent with the broader PAPE literature emphasizing that a conditioning activity can acutely enhance explosive performance, but the magnitude and reliability depend strongly on the characteristics of the conditioning stimulus (e.g., intensity and the neuromuscular demand imposed by the task), rather than movement similarity alone [5,16,23]. In support, Papla et al. compared bilateral and unilateral conditioning activities and reported no meaningful improvement in CMJ, underscoring that exercise selection alone may be insufficient to elicit measurable PAPE [12]. In contrast, Lowery et al. demonstrated that moderate-to-high-intensity squatting can potentiate jump performance and that higher intensity can extend the time window over which potentiation is detectable [16]. Within this framework, the present findings suggest that a split squat-based CA can elicit potentiation in jump outcomes, but the presence of adequate external load appears to be a key factor for translating the CA stimulus into a measurable improvement in CMJ beyond baseline [3,14,16].

### 4.2. Load vs. Unload: Physiological Specificity

The loaded condition (BOSU at 50% 1RM) produced a global increase in neuromuscular demand, evidenced by higher EMG amplitude across hamstrings, quadriceps, gastrocnemius, and stabilization muscles relative to unloaded conditions. The whole-limb recruitment pattern provides a mechanistic basis for the observed enhancement in CMJ performance. Explosive tasks such as SLJ and CMJ require coordinated force production across the hip–knee–ankle flexor/extensor chain, and external loading likely increased neural drive and motor unit recruitment across multiple muscle groups rather than selectively altering activation within a single muscle region [3,5,16]. In line with PAPE mechanisms, this may enhance rate of force development and force transmission efficiency. From a task-specific perspective, the loaded condition likely increased the mechanical demand across both eccentric and concentric phases, facilitating more effective stretch-shortening cycle (SSC) utilization during the subsequent CMJ. Importantly, the selected load represented the highest tolerable intensity that could be executed on BOSU with minimal technical failure, providing a strong yet feasible conditioning stimulus. In contrast, the unloaded conditions appear insufficient to induce a comparable level of neuromuscular potentiation despite similar movement patterns. Interestingly, gastrocnemius medialis did not exhibit the same magnitude or pattern of activation change as proximal muscles. This discrepancy may be explained by its biarticular function and its role within the distal kinetic chain. While proximal muscles (e.g., hamstrings and quadriceps) primarily contribute to force generation and transfer, gastrocnemius medialis plays a more prominent role in ankle plantarflexion and joint stabilization. Under unstable and loaded conditions, its activation may be constrained by the need to maintain balance rather than maximize force output. Additionally, previous studies have suggested that plantar flexors such as gastrocnemius and soleus may exhibit less pronounced or delayed potentiation responses compared to proximal musculature due to their functional role in postural control [18,20]. Taken together, these findings indicate that external load is the primary factor driving CMJ potentiation through increased global neuromuscular activation and SSC efficiency, whereas distal muscles such as gastrocnemius may exhibit more task-dependent activation patterns.

### 4.3. Unstable Surface: Localized Redistribution Rather than Performance Gain

A mechanistically informative outcome was that instability unloaded did not enhance jump performance beyond SS, yet it selectively increased hamstring activity across the split squat cycle, including higher phase-specific AUC in both descending and ascending phases. This indicates that foot instability can redistribute recruitment toward the posterior chain without necessarily increasing net explosive output. Such a pattern is consistent with reports that instability devices during unilateral squat tasks can modify muscle activation even under body-weight conditions [11]. Notably, this redistribution was not uniformly observed across lower-limb muscles including vastus lateralis/medialis and gastrocnemius lateralis/medialis. This absence of change likely reflects the task-specific role of these muscles, which primarily contribute to sagittal plane force production rather than mediating balance-related adjustments. Since the instability in the current study was introduced primarily through foot support rather than perturbing the center of mass substantially, the mechanical demand on knee extensors and ankle plantar flexors remained relatively unchanged. Previous work has similarly reported that instability does not necessarily increase activation of prime movers when balance-relevant joint moments are not substantially altered [18,20]. In addition, our data did not show that a surface change increases EMG activation in gluteus medius or peroneus longus, contrasting with studies where BOSU type platforms increased hip and ankle stabilizer activity during functional tasks [18,20]. Rather than being contradictory, these findings highlight a specific boundary condition: the neuromuscular consequence of instability depends on how it is implemented (rear foot instability, BOSU orientation (up or down) and task constraints) and on how EMG is quantified (cycle-based AUC vs. peak measures). In practical terms, the present results help clarify why unstable surface training sometimes changes muscle activation without improving jump performance: instability may increase control demands and alter coordination strategies (in the current study, greater hamstring contribution) while leaving overall explosive output unchanged unless accompanied by sufficient external loading. In addition to these muscle-specific changes, unstable surface conditions are also known to enhance proprioceptive input and postural control mechanisms [29]. Increased afferent feedback from joint mechanoreceptors and plantar cutaneous receptors under unstable support conditions can elevate the demand for sensorimotor integration and joint stabilization strategies [30]. This may contribute to the observed redistribution toward posterior chain activation, as hamstring muscles play a critical role in controlling knee and hip stability during perturbation [31]. However, such proprioceptive-driven adaptations primarily reflect improved control rather than increased force-generating capacity, which may explain the absence of additional improvements in jump performance despite altered neuromuscular activation.

### 4.4. Limitations

Several limitations should be acknowledged. First, the design did not include a loaded stable surface split squat condition, which prevents complete isolation of load and surface effects; however, the current study’s aim was to test a field-feasible unstable-surface CA model, and the inclusion of both unloaded SS and unloaded BOSU still enables a clear comparison of surface change alone while BOSU loaded isolates the added value of external load within the same unstable configuration. Second, participants were recreationally active young men; therefore, the generalizability of the present findings is restricted to this specific population. Caution is warranted when extrapolating these results to female athletes, older adults, or highly trained populations as neuromuscular responses to conditioning activities may differ across sex, age, and training status. Third, outcomes were limited to jump performance and surface EMG without concurrent kinematics or joint kinetic analyses, which restricts a more comprehensive biomechanical interpretation. Additionally, EMG measurements were limited to the dominant leg, and muscle activity of the rear leg was not assessed. This may limit the understanding of bilateral neuromuscular contributions during the split squat task. Despite these limitations, the present study provides practical insight into how surface instability and external load differentially influence performance and neuromuscular activation within a field-applicable split squat conditioning framework.

## 5. Conclusions

In recreationally active men, split squat conditioning activities produced acute improvements in both CMJ and SLJ; however, external loading was the primary determinant of meaningful performance potentiation. Adding load on BOSU (50% split squat 1RM) elicited the largest CMJ increase and exceeded both unloaded stable surface and unloaded BOSU conditions, whereas a surface change without load yielded comparable jump outcomes. EMG analyses revealed a dissociation between mechanisms: BOSU unloaded selectively increased hamstring activation (biceps femoris and semitendinosus) while BOSU loaded induced a global increase in activation across the hamstrings, quadriceps, gastrocnemius, and stabilizer muscles. From a practical perspective, coaches and practitioners aiming to acutely enhance jump performance should prioritize moderate external loading during unilateral conditioning activities rather than relying solely on surface instability. In contrast, unstable surface conditions without load may be strategically used to target posterior chain activation and neuromuscular control when performance enhancement is not the primary goal. Collectively, these findings indicate that load manipulation is more effective than surface manipulation for enhancing acute jump performance while instability primarily reshapes neuromuscular recruitment strategy. Future studies incorporating loaded stable surface comparisons and mechanical measures (kinematics/kinetics) are warranted to further isolate load/surface interactions and their transfer to sport-specific tasks.

## Data Availability

The original contributions presented in this study are included in the article. Further inquiries can be directed to the corresponding author.
